# Sex-specific differences in transcriptome profiles of brain and muscle tissue of the tropical gar

**DOI:** 10.1186/s12864-017-3652-3

**Published:** 2017-04-07

**Authors:** Kayla M. Cribbin, Corey R. Quackenbush, Kyle Taylor, Lenin Arias-Rodriguez, Joanna L. Kelley

**Affiliations:** 1 School of Biological Sciences, Washington State University, Pullman, WA 99164 USA; 2 División Académica de Ciencias Biológicas, Universidad Juárez Autónoma de Tabasco (UJAT), C.P. 86150, Villahermosa, Tabasco Mexico

**Keywords:** *Atractosteus tropicus*, Tropical gar, Differential expression, Transcriptome assembly, Early vertebrates

## Abstract

**Background:**

The tropical gar (*Atractosteus tropicus*) is the southernmost species of the seven extant species of gar fishes in the world. In Mexico and Central America, the species is an important food source due to its nutritional quality and low price. Despite its regional importance and increasing concerns about overexploitation and habitat degradation, basic genetic information on the tropical gar is lacking. Determining genetic information on the tropical gar is important for the sustainable management of wild populations, implementation of best practices in aquaculture settings, evolutionary studies of ancient lineages, and an understanding of sex-specific gene expression. In this study, the transcriptome of the tropical gar was sequenced and assembled *de novo* using tissues from three males and three females using Illumina sequencing technology. Sex-specific and highly differentially expressed transcripts in brain and muscle tissues between adult males and females were subsequently identified.

**Results:**

The transcriptome was assembled *de novo* resulting in 80,611 transcripts with a contig N50 of 3,355 base pairs and over 168 kilobases in total length. Male muscle, brain, and gonad as well as female muscle and brain were included in the assembly. The assembled transcriptome was annotated to identify the putative function of expressed transcripts using Trinotate and SwissProt, a database of well-annotated proteins. The brain and muscle datasets were then aligned to the assembled transcriptome to identify transcripts that were differentially expressed between males and females. The contrast between male and female brain identified 109 transcripts from 106 genes that were significantly differentially expressed. In the muscle comparison, 82 transcripts from 80 genes were identified with evidence for significant differential expression. Almost all genes identified as differentially expressed were sex-specific. The differentially expressed transcripts were enriched for genes involved in cellular functioning, signaling, immune response, and tissue-specific functions.

**Conclusions:**

This study identified differentially expressed transcripts between male and female gar in muscle and brain tissue. The majority of differentially expressed transcripts had sex-specific expression. Expanding on these findings to other developmental stages, populations, and species may lead to the identification of genetic factors contributing to the skewed sex ratio seen in the tropical gar and of sex-specific differences in expression in other species. Finally, the transcriptome assembly will open future research avenues on tropical gar development, cell function, environmental resistance, and evolution in the context of other early vertebrates.

**Electronic supplementary material:**

The online version of this article (doi:10.1186/s12864-017-3652-3) contains supplementary material, which is available to authorized users.

## Background

The tropical gar fish or pejelagarto (*Atractosteus tropicus*) is a ray-finned chordate that inhabits the tropical freshwater habitats of the Caribbean and Pacific drainages, ranging from Southern Mexico to Northern Costa Rica [1–3]. The tropical gar is one of seven extant species that belong to the family Lepisosteidae, which consists of two genera of non-teleost bony fishes, *Lepisosteus* and *Atractosteus,* which diverged 100 million years ago [4]. The Lepisosteidae family of fishes are often referred to as “living fossils” because they belong to an ancient lineage in which most species are now extinct and extant species have experienced little evolutionary change for the past 100 million years [5]. The tropical gar is distinguished from other gars by its characteristic spotted, long, narrow body and snout, and average mature size of 50–60 centimeters [6]. Their preferred habitat is the slow moving waters of rivers and lakes, as well as backwaters and lagoons. They can survive in low oxygen levels and withstand moderately high water temperatures. The tropical gar is piscivorous [7], and reproduction occurs from March to November and peaks in July and August [8, 9]. In Mexico and Central America, there is a recreational fishing industry for tropical gar, and it is a popular food source due to its nutritional quality and low price. Tropical gar is one of the five main fishery resources in Mexico [1–3].

Because of the regional importance of the tropical gar, concerns about the wild population have arisen. Wild capture reached its peak in 1996, at 530.6 tons. However, in 1999, only 219 tons were captured [10]. No further analyses have been done to determine whether the decrease was due to population decline. The tropical gar has not been evaluated for conservation status by the International Union for Conservation of Nature (IUCN) Red List. Little is known about the species despite concerns about overexploitation by fisheries and habitat degradation caused by dam construction, oil extraction, urban expansion and agricultural expansion [9, 10]. Only one country, Costa Rica, has listed them as endangered [6]. Because of these concerns, several local agencies (for example, the Universidad Juárez Autónoma de Tabasco (UJAT), the Consejo Nacional de Ciencia y Tecnologia (CONACyT) and the Secretaría de Agricultura, Ganadería, Desarrollo Rural, Pesca y Alimentación (SAGARPA)) in Mexico and international agencies (for example, the United Nations Development Programme) are working to raise awareness and educate the public on ways to preserve their native aquatic resources [9, 11].

Interest in tropical gar aquaculture has increased to meet rising demand and to reduce pressure on wild populations. A barrier to breeding gar for food or research is that there is no easy way to differentiate males and females externally [9]. The challenge of distinguishing males from females is most accentuated during early and juvenile stages, whereas adult females are largely identifiable during the reproductive season by their prominent abdomen due to mature ovary development [8]. The only way to definitively distinguish males and females is through invasive procedures to identify whether an individual has ovaries or gonads. Additionally, varying ratios of females to males have been observed, with ratios of females to males as skewed as 1:10 in aquaculture settings [8, 9]. The genetic basis of sex determination in tropical gars has been inconclusive thus far due to the lack of differences in chromosome structure from karyotyping [12, 13] and the overall lack of genetic analyses. However, the skewed sex ratios may be due to an environmental sex determination mechanism, such as temperature-dependent sex determination, which has been identified as an important factor in determining sex ratios in many other species of fishes, amphibians, and reptiles [14].

In this study we examine sex-specific gene expression differences in adult tropical gar. Sex-specific differences have become increasingly evident across species and tissue types [15–18]. Examining these sex differences at a molecular level is important in understanding structural, behavioral, and cellular differences between sexes. Additionally, these expression differences can lead to skewed disease risk between sexes [19]. Differences in gene expression between sexes has been shown to be relevant in human neurological diseases [15] and immune diseases, such as irritable bowel syndrome and allergy [20]. While gene expression differences between sexes can contribute to disease, there is also evidence that sexually dimorphic gene expression patterns are evolutionarily conserved and therefore also important to phenotypic differences between sexes [21].

In this study, the tropical gar transcriptome was assembled *de novo*. The assembled transcriptome was compared to the transcriptome of the spotted gar [22] and was also functionally annotated using existing databases. Ultimately, expression levels between muscle and brain tissue from three male and three female tropical gar were compared to identify sex-specific and highly differentiated transcripts.

## Methods

### Sample material

Muscle, brain, and gonad tissues were dissected from three male and three female tropical gar (*Atractosteus tropicus*) from the Genetic Nucleus of the Tropical Garfish in Tabasco, which is on the campus of the Division for Biological Sciences of the Universidad Juárez Autónoma de Tabasco, Mexico. Fish were fed twice daily with 3.5 millimeter, 32% protein tilapia pellets (Silver Cup-El Pedregal®) and were provided with live tilapia juveniles in their rearing tanks. Rearing tanks were kept at a temperature of 30 ± 0.5°C in a closed room with natural photoperiod. Fish were sacrificed on July 5, 2013, near the beginning of their reproductive season (26 months old) around 11:00 AM by an overdose of Tricaine methanesulfonate (1 gram/5 liters). Tissue samples were dissected and transferred to sterile tubes with fresh RNAlater (Thermo Fisher Scientific #AM7020), which was changed after one hour and the following day. The samples were then kept at -20°C.

### Library preparation and sequencing

Each tissue was disrupted and homogenized by placing the sample in a CryoPrep tissueTUBE (Covaris #520071), freezing the sample in liquid nitrogen and then smashing with a mallet or using 2 millimeter tubes with Lysing Matrix D (1.4 mm) ceramic spheres (MP Biomedicals #116913050) on the Mini-Beadbeater-16 (BioSpec Products #607). Total RNA was isolated from the lysate using either the RNeasy Plus Mini Kit (Qiagen, #74104) or Nucleospin RNA kit (Macherey-Nagel #740698.5) (See Additional file 1: Table S1). The extracted RNA yields were analyzed using the Qubit RNA Assay kit with the Qubit 2.0 fluorometer (Thermo Fisher Scientific, #Q32866). To examine the total RNA quality and concentration, the samples were also analyzed on the 2100 Bioanalyzer system using the RNA Pico Series II kit (Agilent Technologies, #5067-5013).

The mRNA was isolated from the total RNA of each sample using the NEBNext® Poly(A) mRNA Magnetic Isolation Module (NEB, #E7490). Isolated mRNA was further prepared for Illumina sequencing by fragmenting the mRNA, synthesizing double-stranded cDNA, dA tailing, ligating adaptors, and PCR enrichment using the NEBNext® Ultra Directional RNA Prep Kit for Illumina (NEB, #E7420S). Each sample was given a unique barcode for identification. The cDNA was amplified for 13–14 PCR cycles and checked for a visual PCR product using an E-Gel (Thermo Fisher Scientific, #G501802) (Additional file 1: Table S1). The library concentrations were measured with the Qubit dsDNA HS Assay Kit (Thermo Fisher Scientific, #Q32854) and the Fragment Analyzer using a High Sensitivity NGS Fragment Analysis Kit (Advanced Analytical, #DNF-486-0500) or the 2100 Bioanalyzer system using the High Sensitivity DNA Analysis kit (Agilent Technologies, #5067-4626). Libraries were pooled in equal molar concentration (2.5 nmol/L), concentrated to 10 nmol/L, and sequenced on one lane of an Illumina HiSeq 2500 using a 100 base pair paired-end approach at the Genomics Core at Washington State University, Spokane.

### De novo Transcriptome Assembly and Annotation

Raw reads were filtered prior to *de novo* transcriptome assembly. Trim Galore! (version 0.3.7) with FastQC (version 0.11.2) was used to for quality trimming and to trim the standard Illumina adaptors from the sequence data. Twelve base pairs were trimmed from the 5’ end of the reads to avoid base composition biases. For the filtering parameters, the minimum read length was set to 50 base pairs, stringency of 6, quality to 20, and paired-end sequencing was indicated.

Trinity (version 2.1.1) [23, 24] was used to assemble the reads into transcripts *de novo*. Trinity was used with sequence type set for fastq files, library type reverse-forward, maximum memory of 50G, and minimum contig length of 300 base pairs. Scripts distributed with Trinity were used to analyze the assembly and to report the number of transcripts, components, and contig N50 value, which is the maximum length whereby at least 50% of the total assembled sequence resides in contigs of that length or longer. Bowtie2 (version 2.2.6) [25] was used to realign all of the original reads to the Trinity assembled transcripts and RSEM (version 1.2.19) [26] was used to obtain abundance estimates for transcripts based on the number of reads that aligned back to each transcript. Scripts distributed by Trinity were then used to filter out transcripts with an abundance below 0.5 fragments per kilobase transcript length per million fragments mapped (FPKM).

The assembly was compared to the spotted gar fish (*Lepisosteus oculatus*) peptide annotation from the genome assembly by downloading the peptide file from the Ensembl database (GCA_000242695.1). BLAST+ (version 2.2.27) [27] was used to count the number of the tropical gar transcripts aligning to known spotted gar transcripts using an e-value of 10^-5^. Trinotate (version 2.0.1) [23], a comprehensive annotation suite, was used to annotate the assembled transcriptome using an e-value of 10^-5^ in order to identify putative transcript functions. Annotation tools included TransDecoder [23] to predict coding regions in transcripts, HMMER [28] and PFAM [29] for protein domain identification, signalP [30] to predict signal peptides, tmHMM [31] to predict transmembrane regions and the SwissProt database to compare with well-annotated proteins and to retrieve gene ontology (GO) terms [32–34].

### Differential Expression Analysis

Bowtie2 [25] was used to realign the original reads to the Trinity assembled transcripts and RSEM was used to obtain abundance estimates for each sample [26]. EdgeR (version 3.12.0) [35–38] was then used to identify differentially expressed transcripts between male and female brain and muscle samples. Differentially expressed transcripts between males and females in each sample were identified using a False Discovery Rate of less than 5% and log_2_ fold change of 1. Annotations for differentially expressed transcripts were pulled from the Trinotate annotation set. GO enrichment of the differentially expressed transcripts to identify under-represented and over-represented terms in the male and female brain and muscle tissues was analyzed using scripts developed as a part of the Trinity package using a p-value of 0.05 [23, 24].

## Results

### Sequencing and de novo assembly

Muscle, brain, and gonad tissue from three male and female specimens were included in the study. RNA was extracted from all samples. However, after several unsuccessful extractions from female gonad, the tissue was excluded from the experiment. Male gonad, muscle, and brain and female muscle and brain were used for the transcriptome assembly and annotation, but only muscle and brain were used to examine differential expression between males and females. RNA from the samples was prepared into sequencing libraries and sequenced. The sequence data was trimmed, resulting in 510,712,268 reads (Table 1). The reads were assembled into 320,271 putative transcripts using Trinity [24]. After filtering transcripts with a FPKM of less than 0.5, 80,611 transcripts remained, of those 38,146 had predicted open reading frames. The contig N50 was 3,151 base pairs, with over 110 kilobases in the transcriptome assembly, when considering the longest transcript per locus. Transcripts ranged in length from 301–37,561 base pairs. Transcripts from the tropical gar transcriptome assembly were compared to the spotted gar transcriptome using BLAST [27] and 49,994 transcripts from our transcriptome assembly aligned to 15,234 of the 22,483 spotted gar peptide sequences with an e-value of 10^-5^. The spotted gar transcriptome was also compared to the tropical gar transcriptome using BLAST with an e-value of 10^-5^ and 21,887 of the spotted gar peptide sequences aligned to 14,766 tropical gar transcripts. Comparison to the SwissProt database using BLAST with an e-value of 10^-5^ revealed that 47,482 of the tropical gar transcripts had significant hits to 17,732 proteins in the SwissProt database. Over 10,000 of the top hit SwissProt transcripts had at least 80% length coverage (Additional file 1: Tables S2 and S3). Trinotate [24] was used to annotate the assembled transcripts by comparing the *de novo* transcriptome assembly to SwissProt and by running several domain prediction algorithms (see Methods) to determine putative functional domains. Over 59% of transcripts had annotations.

**Table 1:** Number of trimmed reads per sample

Tissue	Individual	Number of Reads
Muscle	Male 1	30,873,758
Brain	Male 1	31,847,458
Gonad	Male 1	27,594,222
Muscle	Female 1	32,480,120
Brain	Female 1	31,980,502
Muscle	Male 2	28,655,062
Brain	Male 2	86,112,076
Gonad	Male 2	21,194,850
Muscle	Female 2	24,367,738
Brain	Female 2	36,943,364
Muscle	Male 3	36,004,898
Brain	Male 3	30,102,592
Gonad	Male 3	34,171,246
Muscle	Female 3	28,281,790
Brain	Female 3	30,102,592

### Differentially Expressed Transcripts

**Fig. 1:**
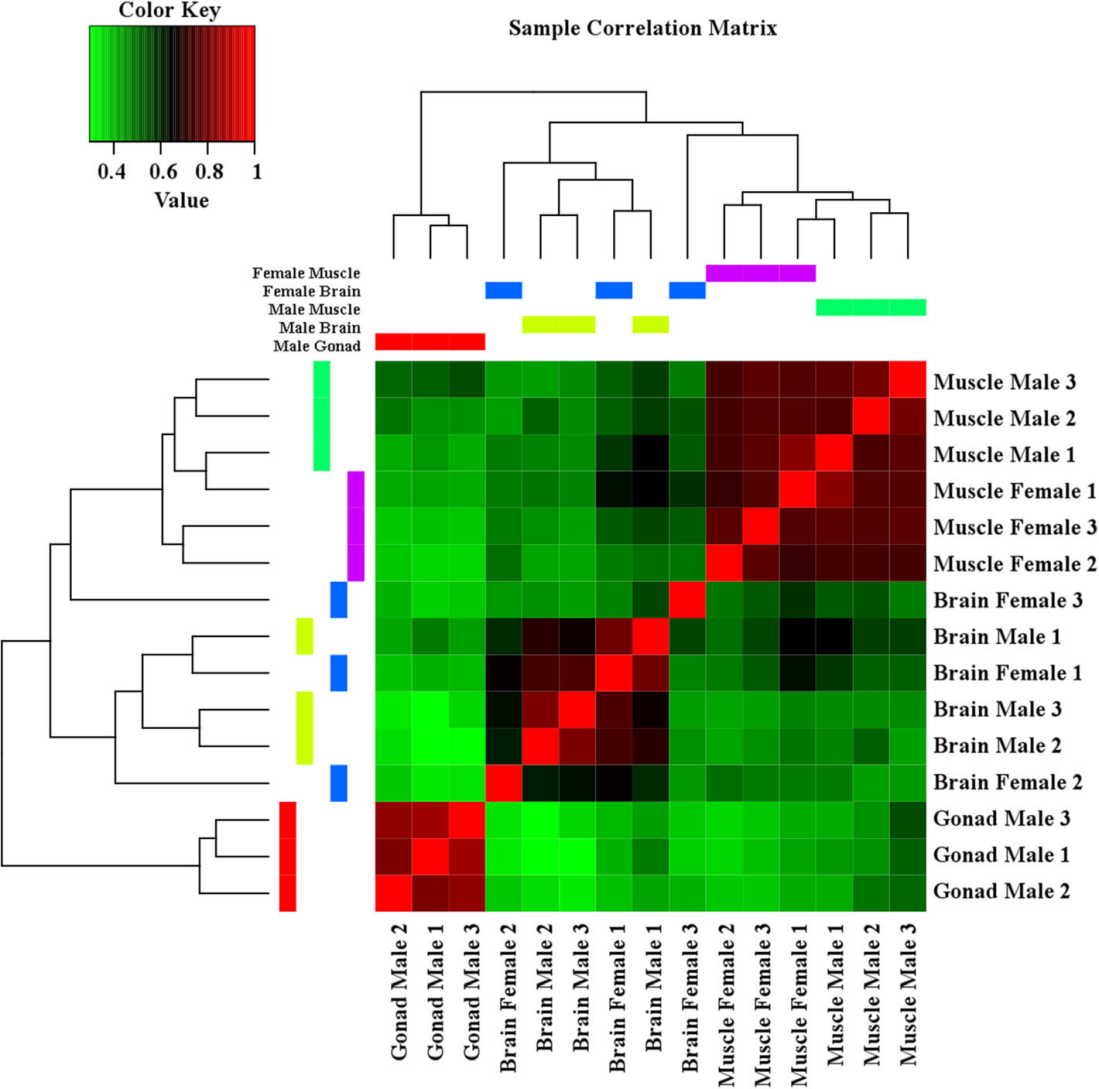
Correlation matrix heat map of transcript expression across all samples

**Fig. 2:**
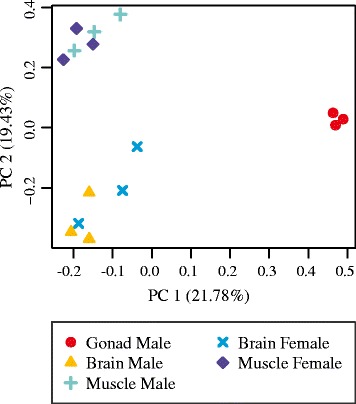
Similarity among the samples based on expression data. Plot of the first two principal components from a PCA analysis of expression data among samples. Percent variation explained by each principal component is in parentheses on each axis. Samples cluster by tissue of origin

**Fig. 3:**
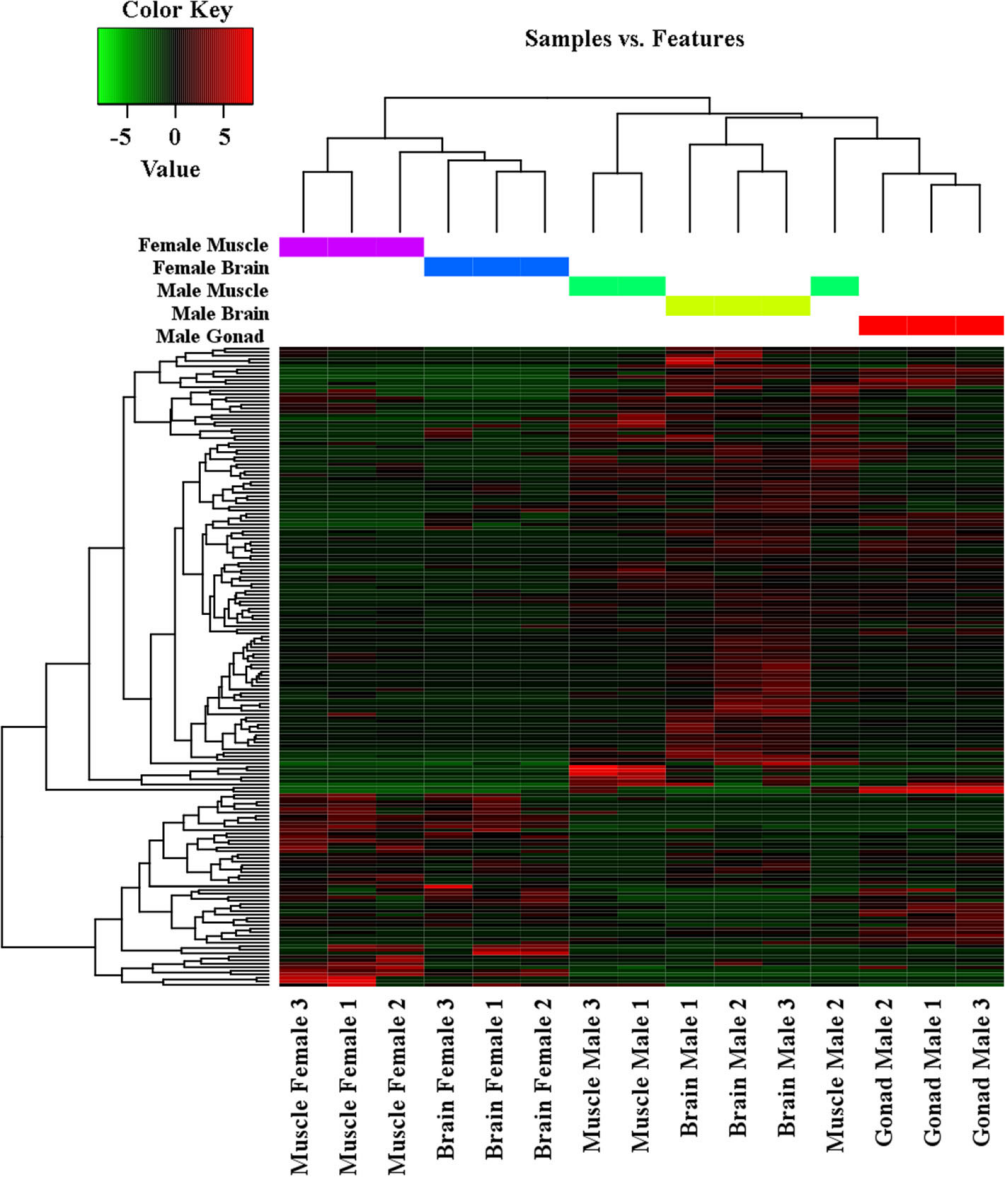
Heat map of differentially expressed transcripts across all samples. Heat map of expression of 109 transcripts in brain tissue and 82 transcripts in muscle identified as significantly differential expressed (FDR < 0.05) and log fold-change of 1 across all samples. *Red* shows high (positive) expression and *green* shows low (negative) expression based on average expression for all samples

To identify differentially expressed transcripts in males and females, brain and muscle samples between the sexes were compared. The samples largely cluster by tissue of origin (Figs. 1 and 2). To identify transcripts that were significantly differentially expressed between males and females, a false discovery rate of less than 0.05 and log_2_ fold change of 1 was used. In the differential expression analysis, 109 transcripts, corresponding to 106 genes, were identified as significantly differentially expressed between the male and female brain samples (Additional file 1: Tables S4-S7; Additional file 2: Figure S1) and 82 transcripts, corresponding to 80 genes, were identified as significantly differentially expressed between the male and female muscle samples (Additional file 1: Tables S8-S11; Additional file 3: Figure S2). A heat map of expression values for the differentially expressed loci shows groups of genes with similar expression patterns, with expression grouping primarily by sex, rather than tissue (Fig. 3). In the brain, 87 of these transcripts were upregulated in the male samples (Additional file 1: Tables S4 and S5) and 22 were upregulated in the female samples (Additional file 1: Tables S6 and S7). In the muscle, 50 of the differentially expressed transcripts were upregulated in the male (Additional file 1: Tables S8 and S9) and 32 were upregulated in the female (Additional file 1: Tables S10 and S11).

### Differential Expression Annotation Analysis

Using Trinotate, transcripts that were identified as differentially expressed were annotated [24]. In the brain tissues, 90% of male and 82% of female significantly differentially expressed transcripts had annotations. In the muscle, 84% of male and 78% of female significantly differentially expressed transcripts had annotations.

In the male brain tissue, upregulated transcripts included those involved in synapses and neuronal cell signaling and regulation, neuronal synaptic plasticity, development, regulation of immune defense response to virus and bacteria, response to stimulus and sensory input, brain development, and mating behavior. There were many transcripts involved in sensory and stimulus response that were significantly differentially expressed in male brain tissue. In the female brain tissue, transcripts that were upregulated were involved in dendrite formation, signaling pathways, cellular response to hypoxia and to viral infection, regulation of circadian rhythm, and response to DNA damage.

Upregulated male muscle transcripts included those involved in mitophagy, viral response, xenophagy, protein autophosphorylation, neuromuscular process controlling balance, protein heterodimerization and homodimerization activity, artery development, and glucose and identical protein binding. In female muscle, upregulated transcripts included those involved in regulation of cell migration, tissue remodeling, response to oxidative stress and hypoxia, myotube differentiation, protein transport, signal transduction and receptor activity, and hormone response.

Gene Ontology enrichment analysis was performed on the differentially expressed transcripts to identify under-represented and over-represented terms. In the male brain, seven terms were identified as under-represented and were involved in cellular metabolic processes and 371 terms were identified as over-represented. Over-represented ontology terms included negative regulation of lamellipodium morphogenesis (GO:2000393), ruffle assembly (GO:1900028), sensory perception of taste (GO:0050909), and mating behavior (GO:0007617) (Additional file 1: Table S12). In the female brain, one term was identified to be under-represented and 177 were identified as over-represented. Over-represented terms included alternative mRNA splicing (GO:0000380), positive regulation of oxidative stress-induced intrinsic apoptotic signaling pathway (GO:1902177), response to oxidative stress (GO:1900409), and negative regulation of circadian rhythm (GO:0042754) (Additional file 1: Table S13). In the male muscle, no terms were found to be under-represented and 223 were over-represented, including terms related to UTP-monosaccharide-1-phosphate uridylyltransferase activity (GO:0003983), glucose binding (GO:000553), and several structural cell components (Additional file 1: Table S14). In the female muscle, one term was identified as under-represented and 221 terms were identified as over-represented. Over-represented terms included negative regulation of Notch signaling pathway (GO:0045746), cellular amino acid metabolic process (GO:0006520), and other protein related terms (Additional file 1: Table S15).

## Discussion

In this study, we assembled and annotated the transcriptome for the tropical gar; the main goal was to examine differential expression between males and females in brain and muscle tissue. Several transcripts were identified as differentially expressed between males and females in these tissues and had varying functional roles. A higher percentage of transcripts were identified as differentially expressed in the brain than in the muscle. Additionally, there were more upregulated transcripts identified in males than females across both tissue types. This sex bias towards more upregulated transcripts in the male is not surprising as other studies have found sex-biased expression in many tissues, with the brain being the second most male-biased tissue after the gonads [39, 40].

In females, some of the upregulated genes in muscle were related to tissue remodeling while in similar male tissues, upregulated genes were involved in mitophagy and other cellular regulating tasks. Overall, many of the transcripts identified as differentially expressed between the male and female muscle tissues were involved in very similar protein related functions. Sex-specific genes include HSPBB (Heat shock protein beta-11; TRINITY_DN121351_c0_g1_i1), which had high expression in all three females sampled and very low expression in the three males, and for example, STOM (Erythrocyte band 7 integral membrane protein; TRINITY_DN131015_c4_g1_i2), was highly expressed and male sex-specific.

Other studies have found similar sex differences in gene expression, indicating that this is a widespread phenomenon in gene expression patterns across organisms. For example, significant differences in expressed levels of certain housekeeping genes commonly used in qPCR have been found between sexes in zebrafish [41]. Sex-specific differences in gene expression have also been detected in many other species, including *Drosophila* [18] and mice [42]. It has been proposed that sex-biased expression may be caused by sexual selection of males by females and/or male gamete competition [17]. In humans, sex differences in gene expression in brain tissue has been found to contribute to differential disease risk between sexes [15]. In rainbow trout, more upregulated genes have been found in male muscle tissue as compared to muscle tissue in females [16]. Moreover, genes with similar functions were identified as highly differentially expressed in female muscle in both the trout study [16] and our study, including acetyl-CoA carboxylase genes (beta (ACACB) in [16] and alpha (ACACA) here) and vacuolar protein sorting-associated proteins (VPS 13A in [16] and VPS 37C here). In examining gene enrichment, GO:0071822 (protein complex subunit organization), GO:0010467 (gene expression), and GO:0019083 (viral transcription) were found to be shared enriched terms in the male samples in both the rainbow trout and tropical gar.

## Conclusions

By identifying, annotating and examining differentially expressed transcripts in the tropical gar transcriptome, we have provided a blueprint for future research into sex-specific gene expression and disentangling the role and mechanism of these differences. The transcriptome also provides a resource for understanding the skewed female-to-male sex ratio in tropical gar. The assembly of the transcriptome of the tropical gar is an important step in gar genomics and adds to the increasing evidence of sex-specific gene expression. The seven extant gar species represent an ancestral clade and an outgroup to teleosts and the teleost whole genome duplication event [22]. This study, combined with recent *de novo* genome assembly of the spotted gar (*Lepisosteus oculatus*) [22, 43], provide the foundation for a better understanding of vertebrate evolution, as well as gar biology, including developmental stages, habitat complexity, resistance, and resilience. Indeed, as a “living fossil,” the tropical gar could prove useful in understanding the early evolution of ancient vertebrate fishes that led to present day diversity.

## Additional files

Additional file 1: **Table S1.** Extraction and library preparation details for each sample. **Table S2.** Top-matching unique BLAST alignments to SwissProt database separated by percent length coverage. **Table S3.** Top-matching unique BLAST alignments to SwissProt database combining multiple high-scoring segment pairs (HSPs). **Table S4.** Transcripts identified as significantly differentially expressed in male and female brain tissues that are upregulated in male brain tissue. **Table S5.** Expression values for transcripts identified as significantly differentially expressed between male and female brain tissues that are upregulated in male brain. **Table S6.** Transcripts identified as significantly differentially expressed in male and female brain tissues that are upregulated in female brain tissue. **Table S7.** Expression values for transcripts identified as significantly differentially expressed between male and female brain tissues that are upregulated in female brain. **Table S8.** Transcripts identified as significantly differentially expressed in male and female muscle tissues that are upregulated in male muscle. **Table S9.** Expression values of transcripts identified as significantly differentially expressed between male and female muscle tissues that are upregulated in male muscle. **Table S10.** Transcripts identified as significantly differentially expressed in male and female muscle tissues that are upregulated in female muscle. **Table S11.** Expression values of transcripts identified as significantly differentially expressed between male and female muscle tissues that are upregulated in female muscle. **Table S12.** Enriched and depleted Gene Ontology (GO) terms for male brain. **Table S13.** Enriched and depleted Gene Ontology (GO) terms for female brain. **Table S14.** Enriched Gene Ontology (GO) terms for male muscle. **Table S15.** Enriched and depleted Gene Ontology (GO) terms for female muscle. (XLSX 150 kb)

Additional file 2: Figure S1. Results from differential expression analysis of female and male brain tissue. **A)** MA plot for each transcript comparing the log_2_ fold-change versus the average transcript expression. Each dot represents a transcript and the significantly differentially expressed (false discovery rate (FDR) < 0.05) transcripts are colored in red. **B)** Volcano plot of FDR as a function of fold change between samples. Significantly differentially expressed (FDR < 0.05) transcripts are colored in red and the FDR threshold is represented as a horizontal orange line. (ZIP 193 kb)

Additional file 3: Figure S2. Results from differential expression analysis of female and male muscle tissue. **A)** MA plot for each transcript comparing the log_2_ fold-change versus the average transcript expression. Each dot represents a transcript and the significantly differentially expressed (FDR < 0.05) transcripts are colored in red. **B)** Volcano plot of FDR as a function of fold change between samples. Significantly differentially expressed (FDR < 0.05) transcripts are colored in red and the FDR threshold is represented as a horizontal orange line. (ZIP 162 kb)

## Abbreviations

ACACA: acetyl-CoA carboxylase alpha
ACACB: acetyl-CoA carboxylase beta
BLAST: Blast local alignment search tool
CONACyT: Consejo Nacional de Ciencia y Tecnologia
FDR: False discovery rate
FPKM: Fragments Per Kilobase of transcript per Million mapped reads
GO: Gene ontology
HSPBB: Heat shock protein beta-11
IUCN: International Union for Conservation of Nature
KEGG: Kyoto encyclopedia of genes and genomes
PCA: Principal component analysis
PCR: Polymerase chain reaction
qPCR: Quantitative polymerase chain reaction
SAGARPA: Secretaría de Agricultura, Ganadería, Desarrollo Rural, Pesca y Alimentación
STOM: Stomatin, Erythrocyte band 7 integral membrane protein
UJAT: Universidad Juárez Autónoma de Tabasco
