# Supplementary figures and images for: Sex-specific differences in transcriptome profiles of brain and muscle tissue of the tropical gar
Source: BMC Genomics. 2017 Apr 7;18:283. doi: 10.1186/s12864-017-3652-3 (PMC5383948; doi:10.1186/s12864-017-3652-3)

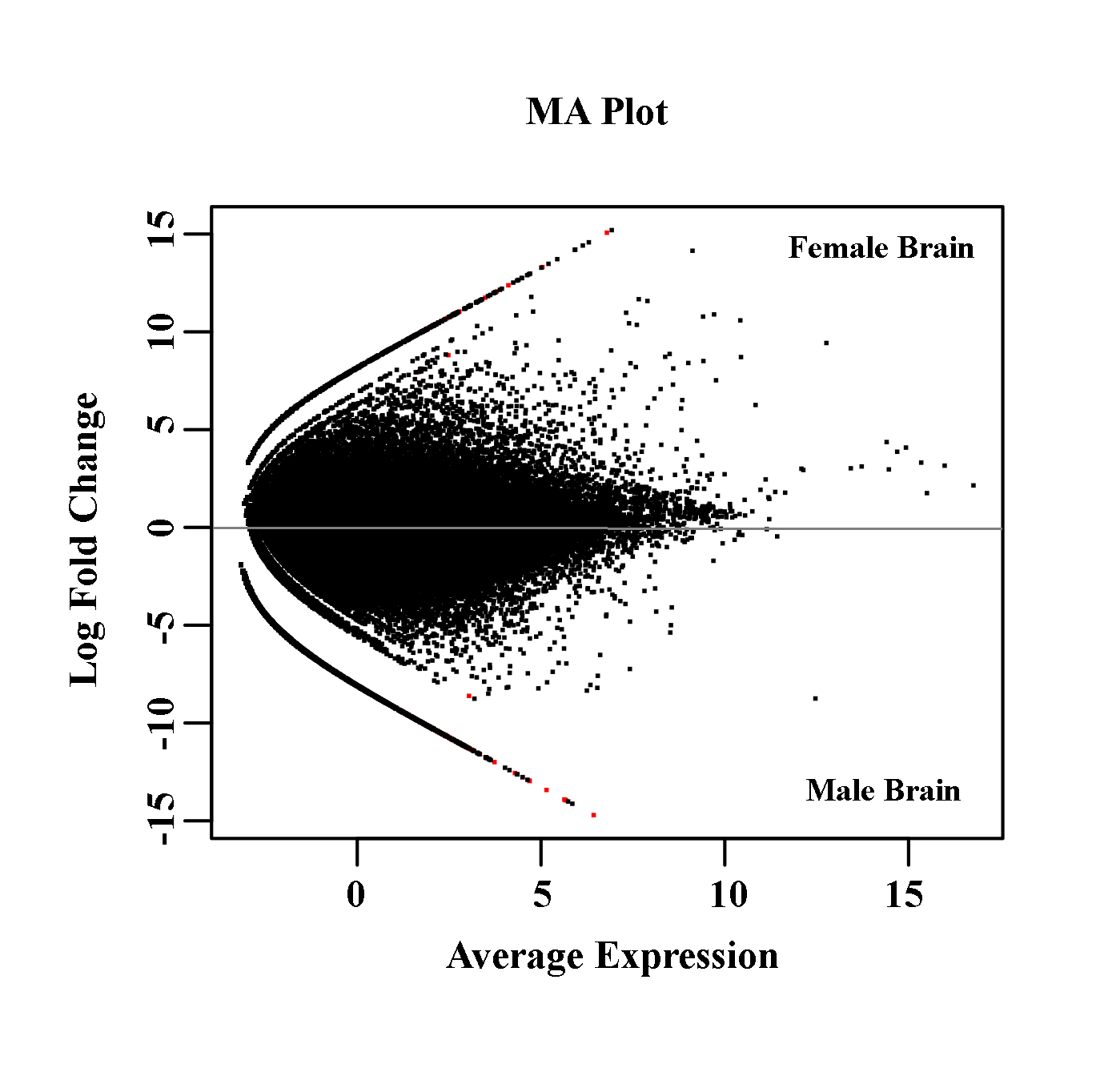

Supplement: Supplementary file 2 — Results from differential expression analysis of female and male brain tissue. A) MA plot for each transcript comparing the log2 fold-change versus the average transcript expression. Each dot represents a transcript and the significantly differentially expressed (false discovery rate (FDR) < 0.05) transcripts are colored in red. B) Volcano plot of FDR as a function of fold change between samples. Significantly differentially expressed (FDR < 0.05) transcripts are colored in red and the FDR threshold is represented as a horizontal orange line. (ZIP 193 kb) [file 12864_2017_3652_MOESM2_ESM.zip › SupplementaryFigure1A.png]

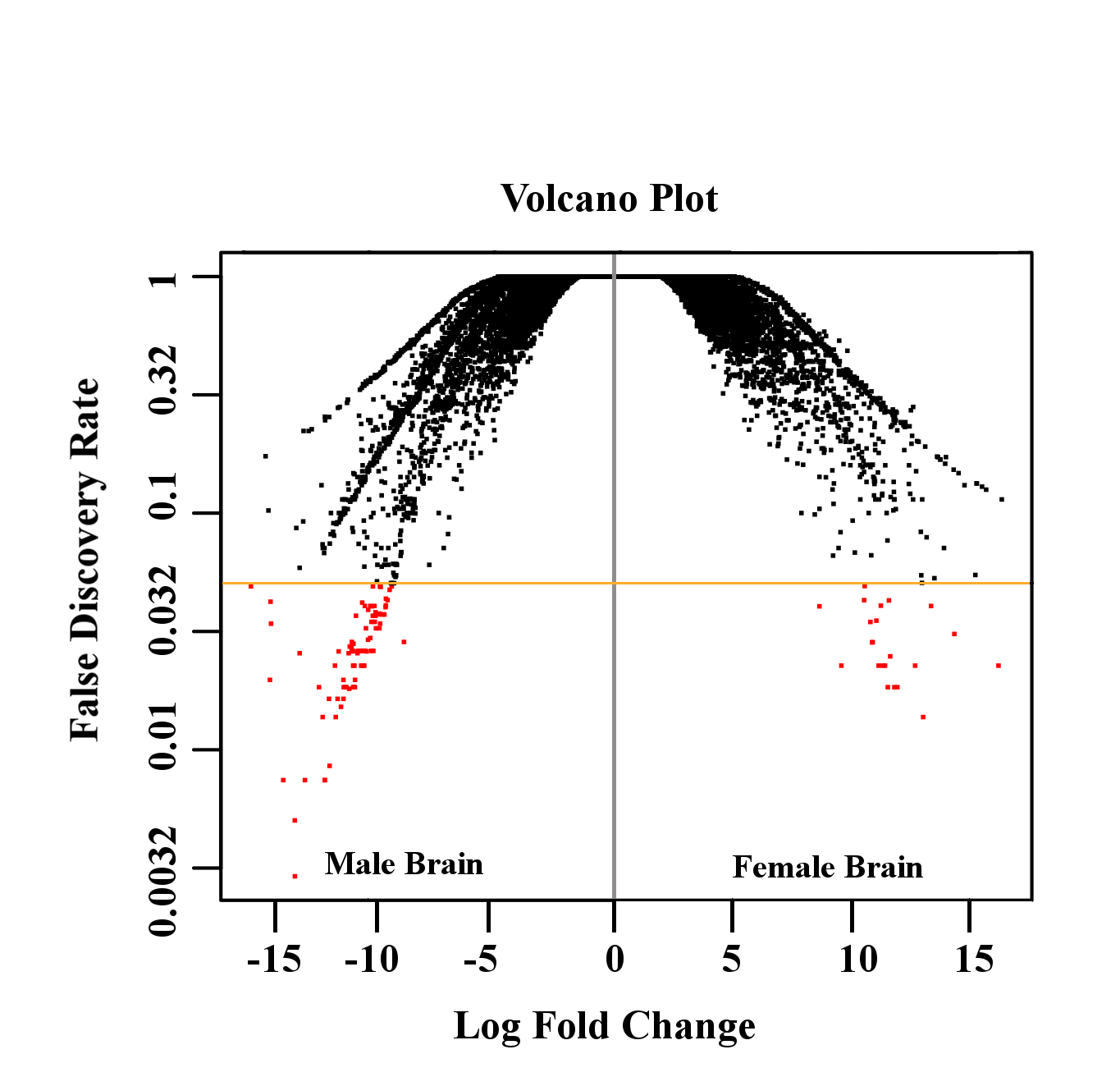

Supplement: Supplementary file 2 — Results from differential expression analysis of female and male brain tissue. A) MA plot for each transcript comparing the log2 fold-change versus the average transcript expression. Each dot represents a transcript and the significantly differentially expressed (false discovery rate (FDR) < 0.05) transcripts are colored in red. B) Volcano plot of FDR as a function of fold change between samples. Significantly differentially expressed (FDR < 0.05) transcripts are colored in red and the FDR threshold is represented as a horizontal orange line. (ZIP 193 kb) [file 12864_2017_3652_MOESM2_ESM.zip › SupplementaryFigure1B.png]

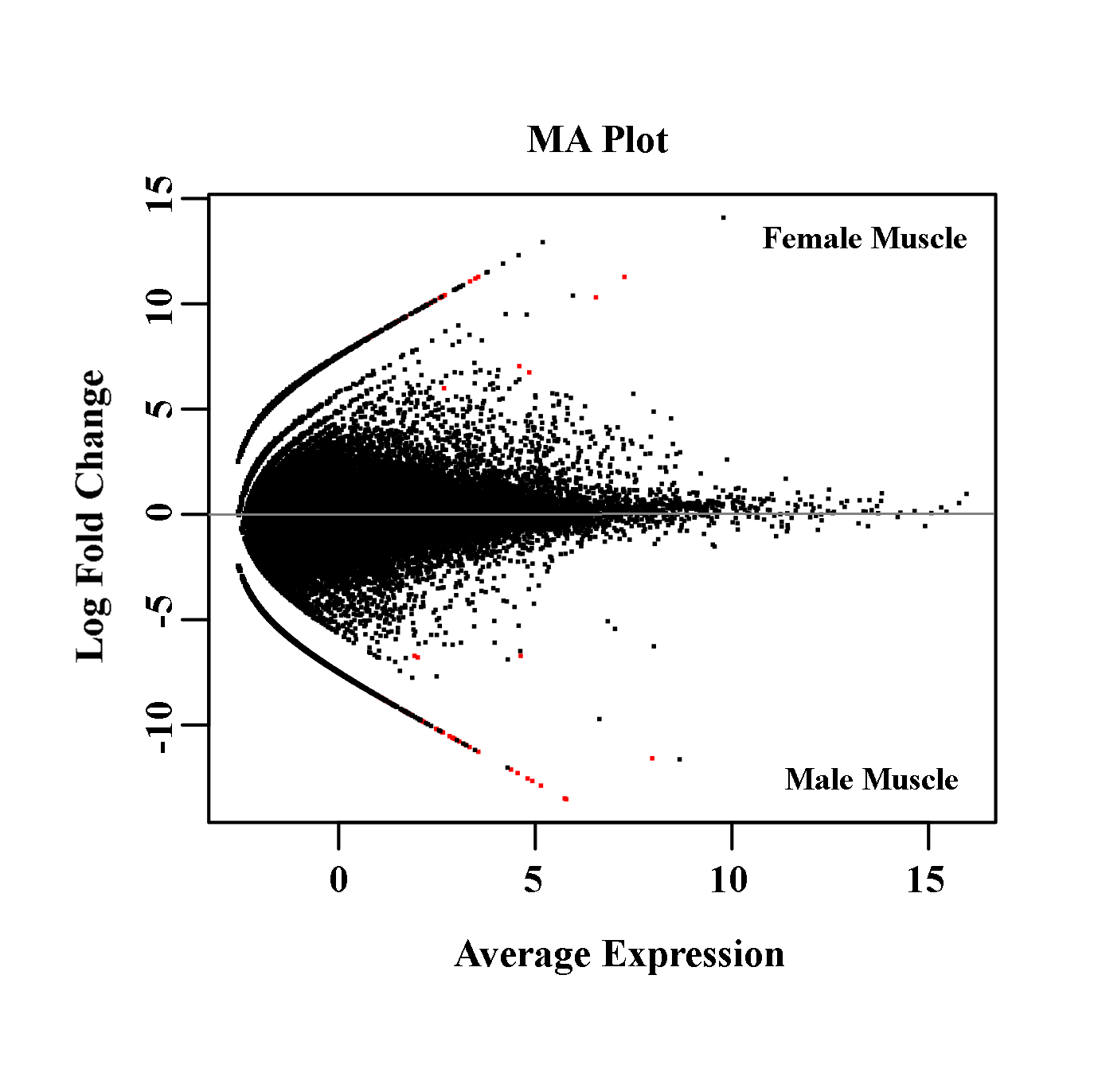

Supplement: Supplementary file 3 — Results from differential expression analysis of female and male muscle tissue. A) MA plot for each transcript comparing the log2 fold-change versus the average transcript expression. Each dot represents a transcript and the significantly differentially expressed (FDR < 0.05) transcripts are colored in red. B) Volcano plot of FDR as a function of fold change between samples. Significantly differentially expressed (FDR < 0.05) transcripts are colored in red and the FDR threshold is represented as a horizontal orange line. (ZIP 162 kb) [file 12864_2017_3652_MOESM3_ESM.zip › SupplementaryFigure2A.png]

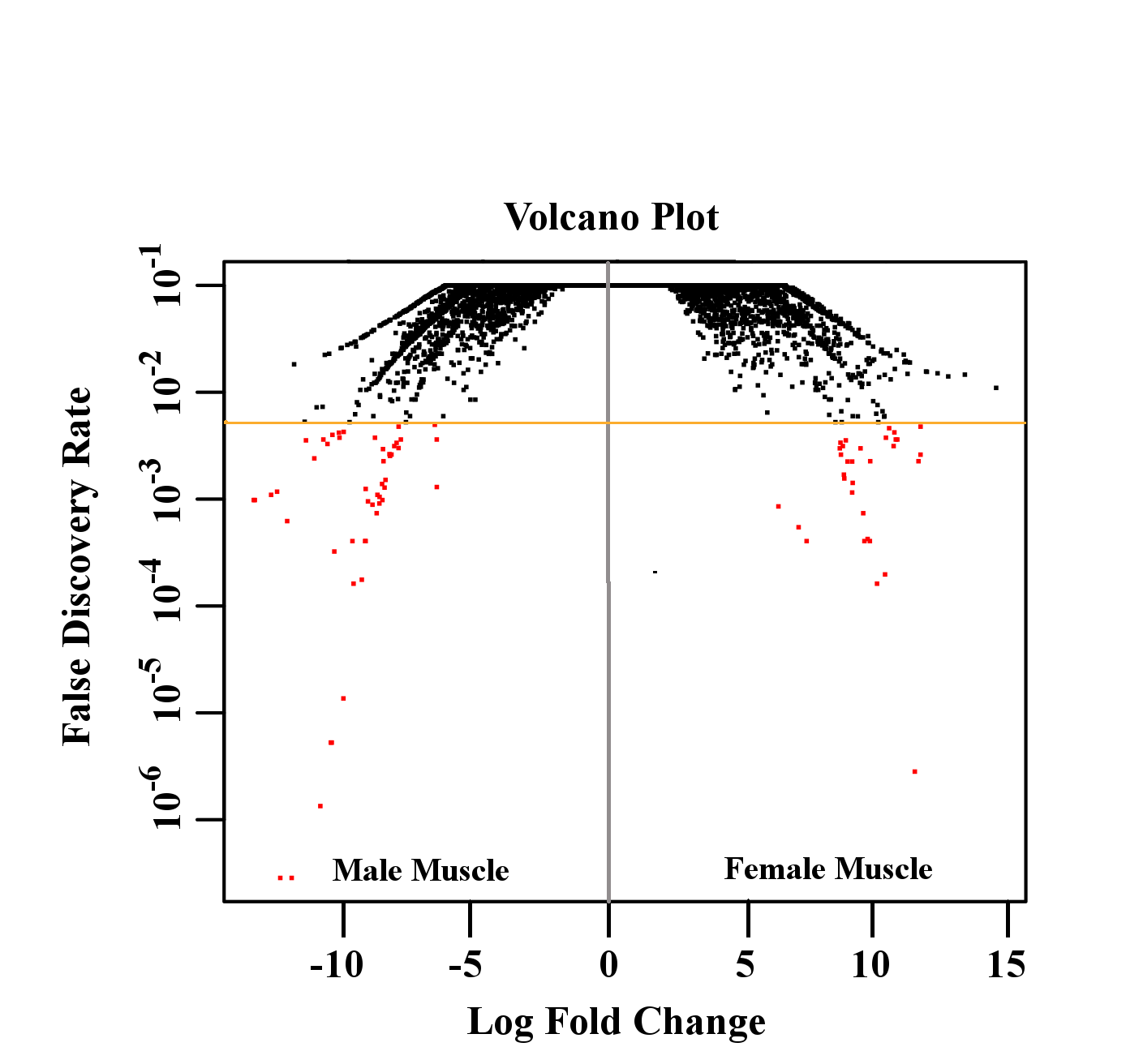

Supplement: Supplementary file 3 — Results from differential expression analysis of female and male muscle tissue. A) MA plot for each transcript comparing the log2 fold-change versus the average transcript expression. Each dot represents a transcript and the significantly differentially expressed (FDR < 0.05) transcripts are colored in red. B) Volcano plot of FDR as a function of fold change between samples. Significantly differentially expressed (FDR < 0.05) transcripts are colored in red and the FDR threshold is represented as a horizontal orange line. (ZIP 162 kb) [file 12864_2017_3652_MOESM3_ESM.zip › SupplementaryFigure2B.png]
